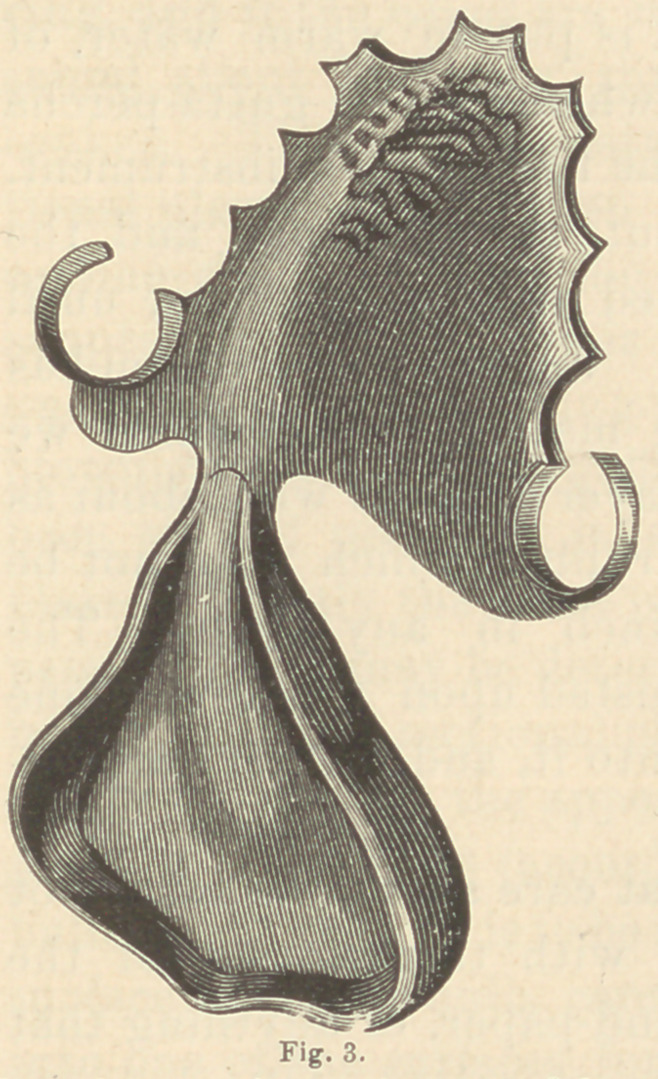# The Construction of Suersen’s Obturators

**Published:** 1885-06

**Authors:** Th. Weber

**Affiliations:** Helsingfors, Finland


### THE CONSTRUCTION OF SUERSEN'S OBTURATORS.

BY DR. TH. WEBER, HELSINGFORS, FINLAND.

(Concluded from page 245, May number.)

When a correct plaster model has been obtained, a plate is made with an appendix on its posterior border extending somewhat into the cleft, to which a strong wire loop is attached. (See fig. 1.)

Fig. 1.

The wire loop upon which the obturator is to be constructed, must not extend further down than the beginning of the uvula. Its direction, when put into the mouth, should be a little above the muscles of the soft palate, in such a manner that when the gutta-percha is applied it will occupy about the centre of the bulb.

If the cleft involves the hard palate, the plate must cover the defect to the lower border of the nasal cavities. The plate should not extend into the nasal cavities, as it may obstruct the free passage of the air through them, in consequence of which the letters M and N would be pronounced indistinctly, and the vowels be accompanied by a nasal sound.

The palate must be held securely in position by at least two well fitting metal clasps, around the molars or bicuspid teeth, if possible. I have constructed one obturator for a patient who had lost all of the teeth from the upper jaw, but I find that it is much more troublesome to become accustomed to an appliance where it cannot be attached to the natural teeth.

When a plate has been constructed as above described, it should be put in the mouth and the patient directed (if time will permit) to wear it for a day. After the plate has been put in position and before the construction of the obturator, we must take care: 1st. That it does not cause pain in any place. 2d. That it does not drop when the wire loop is pressed backwards with the finger. 3d. That the loop occupies its proper position (see fig. 2), after which the construction of the bulb can be commenced.

For this purpose crude gutta-percha has wonderful properties, and is the substance with which Dr. Suersen has achieved his great success in obtaining a correct impression of the muscles of the soft palate and pharynx during articulation, which method was first described by Dr. S. at the meeting of the *Central Verein Deutscher Zahnarzte,* at Hamburg, in 1867. This gutta-percha, after having been warmed, remains for a few hours soft enough to enable the muscles to imbed themselves in it to great perfection, and without discomfort to the patient. In constructing the bulb, the gutta-percha may be warmed over an alcohol lamp, and moulded around the wire loop. The first day only enough gutta-percha should be applied to barely touch the muscles of the soft palate on either side, and the superior constrictor of the pharynx on the posterior part of the bulb. This first layer of gutta-percha must become perfectly hard before another layer is applied, as the action of the muscles may cause the bulb, when modeled too large at the first sitting, to become loosened from the wire loop. The hardening of the first layer, however, if the time is limited, may be accomplished by cooling the apparatus with ice. The amount of gutta-percha to be applied around the loop depends, of course, upon the width of the cleft. The thickness, however, is somewhat definite.

Fig. 2.

Until a few months ago Dr. Suersen, as well as the writer, had made these obturators thick and large, measuring on their posterior part from three-quarters to one inch in thickness, and this, on account of the weight and expansion of the rubber, made it necessary to vulcanize them hollow. While in this country, I have made experiments which have demonstrated that an obturator that is one-eighth of an inch in thickness in its middle part, and three-eighths of an inch high around the borders which are in contact with the muscles, will give the same result as a very thick apparatus. The size of the gutta-percha modeled around the wire loop should, therefore, if a thin obturator is to be made, not exceed one-half an inch in thickness.

When the first portion of the gutta-percha has become hard, another layer may be added, but before this is done it must be carefully dried with a towel and touched with a hot knife. The gutta-percha to be added must be warmed over an alcohol lamp, and with a hot knife modeled into the desired form. If any gutta-percha is to be cut away at any time, the knife must be made sufficiently hot so that it will cut without much pressure. Whenever any gutta-percha has been added to the bulb, as soon as the apparatus has been replaced in the mouth the patient should be directed to swallow, and then read a few lines, or pronounce some letter of the alphabet. Whenever the gutta-percha is in contact with the muscles of the soft palate or pharynx for a few hours, it is marked by them as a polished surface. When, therefore, an obturator has been in position some hours, the places where it is in contact with the muscles may be easily recognized. A rough depression always signifies insufficiency of material. In those places more gutta-percha should be added, until all surfaces which lie in contact with the muscles of the soft palate and pharynx are perfectly smooth.

The lingual surface of a Suersen obturator should be in about the same line as the velum when elevated by the levator palati. The posterior wall of the bulb should always be made to come in contact with the superior constrictor of the pharynx, in that place in which this muscle shows its greatest constriction when the letter A is pronounced. When the apparatus is too long, it is the cause of nausea, and is an obstruction in deglutition; if it is too short, the letters G and K cannot be pronounced perfectly.

In the middle line of the posterior border I have found some advantage in adopting the idea of Dr. Bishop, which consists in forming an imitation of the uvula, as this enables us to make a corresponding excavation at the upper border of the posterior surface, thereby enabling the secretions which collect in the upper surface of the bulb to flow off freely. During articulation the soft palate is continually elevated. It is, therefore, advisable to assure an absolutely tight fit, and the upper border of the lateral walls, which are in contact with the muscles of the soft palate, should be directed a little outwards. For this purpose the bulb across the lingual (lower) surface should be a little narrower than at the same point at the nasal (upper) surface. The posterior surface of the bulb differs in form in almost every case. On the lingual border, about the median line, where the uvula is situated, the upper border should be scalloped out to correspond with the condition presented.

Fig. 3.

The superior surface should be concave, as represented in fig. 3. To assure a perfect adaptation it is best, even when all these surfaces have been worn perfectly smooth, to add a very thin layer of gutta-percha all around the bulb, and then let the patient wear the apparatus for a few days longer. But it should be carefully observed that the posterior wall of the bulb does not extend higher up than the point where the greatest contraction of the superior constrictor muscle is noticeable. In such an instance the pronunciation of the word "mamma" will sound "baba," and the patient will not be able to breathe nor blow freely through the nose. Whenever we have added too much gutta-percha, the bulb should be carefully warmed and the obturator replaced in the mouth. If now the patient is directed to swallow, then blow his nose, and then read a few lines, we will find that the muscles have pushed away the excess of material, which afterwards may be trimmed off with a hot knife. When we are satisfied with the adaptation and position of the obturator, and we are certain that no more alterations are necessary, the gutta-percha bulb may be replaced by either hard rubber or metal. Before placing the obturator in the flask, it is better to secure a correct mould of the same in plaster, that in case anything should happen to the bulb it may not be necessary to commence again with the construction of a new bulb in gutta-percha. This mould must be made out of as many sections as necessary, that the obturator may be removed from the mould with ease. When the obturator has been carefully removed from the sectional mould, it is invested, with the lingual surface downward, in the lower part of a large rubber flask. The gutta-percha bulb should be set into the plaster in such a manner that only its superior surface is exposed. (The plaster in the lower part of the flask should cover every surface of the gutta-percha bulb which has been in contact with any of the muscles of the mouth.) When the plaster is thoroughly hard, and before that for the upper part of the flask is to be poured, the lower part of the flask is put in warm water, of a temperature not higher than 125° F., when all the gutta-percha is to be very carefully removed out of the flask with an instrument. After the gutta-percha has been taken out, the wire loop and the hard rubber appendix should be washed with chloroform, until every trace of gutta-percha is removed. When the chloroform has been allowed to evaporate, we cover the matrix, out of which we have removed the gutta-percha, with a layer of sheet wax, about as thick as we desire to make the bulb of rubber, which need not be thicker than about one-eighth of an inch in any place. The upper part of the flask may then be adjusted upon the lower one thus prepared, and the plaster poured into it, and further manipulated like an ordinary rubber plate.

In finishing the hard rubber bulb, great care should be taken not to touch the sides which lie in contact with the muscles of the soft palate and pharynx with the file, sand-paper, or anything that may remove the contours made by these muscles. All edges, as well as the lingual (lower) and the nasal (upper) surfaces, should be carefully smoothed and polished.

When the obturator is to be made entirely of metal, the plate covering the hard palate with the appendix and wire loop has to be constructed first, as in the case when a rubber plate is desired, when the gutta-percha bulb is moulded in the manner as mentioned above. When the bulb has been constructed, the obturator is imbedded in plaster with the lingual surface downwards, in sections, as mentioned before, the upper surface being left free. When the plaster is perfectly hard the obturator is removed, the sections put together, and thoroughly soaked with a solution of soap in alcohol. This matrix is then filled two or three times, according to the number of metal casts desired, with a mixture of sand and plaster. These pieces of sand and plaster, when removed from the matrix, represent an exact duplicate of the gutta-percha bulb, and are to be fastened upon another large, flat piece of sand and plaster, and gradually but thoroughly dried. When perfectly dry, a strong iron casting-ring, with its widest side downwards, is placed over one of them, and surrounded with casting sand. Into this mould we pour either zinc, type, or Spence-metal, which will give us an exact duplicate of the sectional plaster matrix.

The metal matrix, when cold, is to be removed from the iron ring, and if the edges are much overhanging, the matrix should be sawed about half way through in two or three places, and then broken. This broken matrix, now being an imitation of the sectional plaster one, is then fitted back again into the strong iron casting-ring, into which the metal bulb is to be shaped. The metal best adapted for this purpose is thin, twenty-two carat gold plate, subsequently stiffened by lining it with platinum wire gauze, and then by blowing eighteen carat gold solder over that. When the bulb has been shaped perfectly, it is attached to the appendix of the gold plate in exactly the same position formerly occupied by the gutta-percha, which may be determined by placing both the plate and the bulb in the first sectional plaster mould. When the bulb has been securely soldered to the appendix of the plate, the missing lower (lingual) or upper plate (according to which side was superior when the bulb was struck up) is to be attached. In soldering the upper or lower surface upon the bulb, care should be taken to make a small hole into one of the surfaces for the escape of the air while soldering, which afterwards may be closed by a screw, or by soft solder. The obturator is then, after it has been carefully finished and polished, ready for use.

When these instruments have been carefully constructed in the manner mentioned above, they will give satisfaction in every case, although a great deal of the success depends upon the intelligence and the persistence of the patient. With an acquired lesion there will be very little trouble. The patient has already been taught to articulate, therefore, as soon as the lesion is repaired perfectly, in the majority of cases they can articulate as perfectly as before the perforation of the soft palate. With a congenital defect, on the contrary, the success is never as rapid, for, as mentioned before, these patients do not know how to use the muscles of the tongue, soft palate, and pharynx correctly, to produce perfect articulating sounds. The most difficult letters for such patients to pronounce are S, R, G and T, in combination with other letters, as Sister, Reaping, Coming, Going, Teething, etc., and however perfect the obturator has been constructed, the patient will be unable to acquire articulation without teaching. We should, therefore, see such patients frequently, hear them read slowly, and correct every mistake, by explaining to them what position they should give to the muscles of the tongue to overcome these difficulties; and as daily instruction will be necessary, it will be desirable to have friends of the patient present, that they may become acquainted with the manner of correcting mistakes in articulation.

## Figures and Tables

**Fig. 1. f1:**
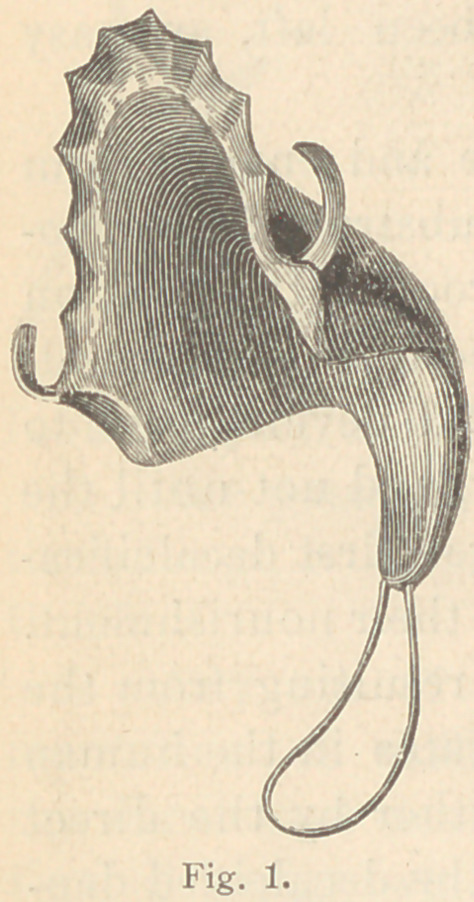


**Fig. 2. f2:**
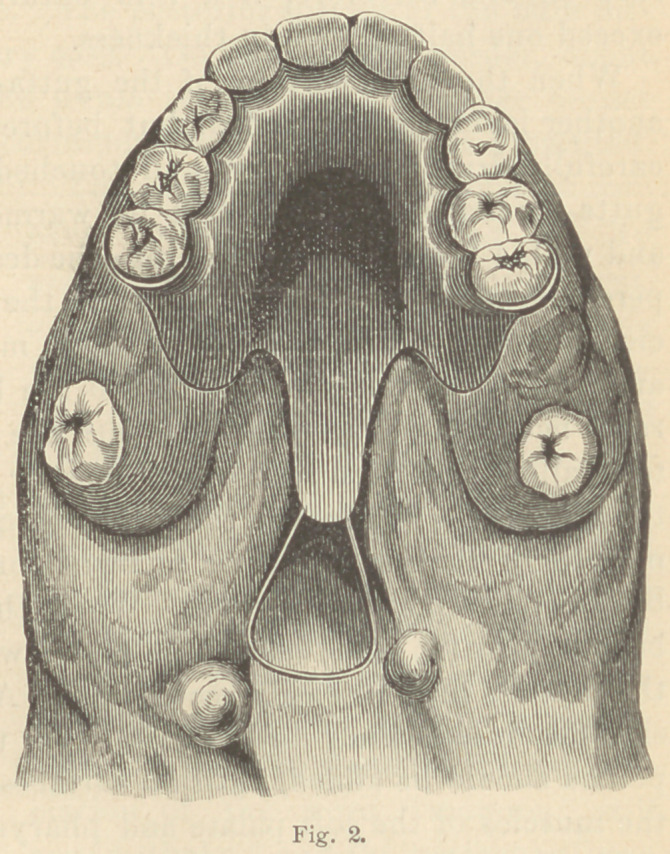


**Fig. 3. f3:**